# SBAR Method for Improving Well-Being in the Internal Medicine Unit: Quasi-Experimental Research

**DOI:** 10.3390/ijerph192416813

**Published:** 2022-12-14

**Authors:** María Cristina Martínez-Fernández, Sara Castiñeiras-Martín, Cristina Liébana-Presa, Elena Fernández-Martínez, Lisa Gomes, Pilar Marques-Sanchez

**Affiliations:** 1 SALBIS Research Group, Faculty of Health Sciences, Universidad de León, 24401 León, Spain; 2 Son Llatzer Hospital, 07001 Palma de Mallorca, Spain; 3 Nursing School, Minho University, 4704-553 Braga, Portugal

**Keywords:** SBAR, handoff or handover, multidisciplinary communication, patient safety, job satisfaction, engagement, resilience, health personnel

## Abstract

SBAR (Situation, Background, Assessment, Recommendation) is a tool for standardizing and improving interprofessional communication. This study aims to explore the impact of SBAR in healthcare professionals’ wellbeing, through concepts such as job satisfaction, engagement, resilience, and job performance, in the internal medicine unit of a university hospital in the province of León (Spain). This is an observational, descriptive, longitudinal case study with a pre- and post-intervention approach. Questionnaires were distributed to a group of doctors, nurses, and healthcare assistants before and after the implementation of the SBAR tool in the ward. The use of SBAR was monitored to ensure staff compliance. Data statistical analysis was performed using the SPSS program. Resilience levels increased significantly post-intervention. Job satisfaction and engagement levels remained neutral, slightly decreasing post-intervention. Besides’ being a useful tool to improve communication, SBAR was effective in improving resilience among staff. Several aspects related to hospital management may have had an impact on job satisfaction and engagement results.

## 1. Introduction

SBAR is a widely used and recommended tool that has proven useful in improving interprofessional communication, a key element in healthcare [1,2]. This method has been recommended by numerous organizations, such as the Canadian Patient Safety Institute [3], or in the OSSIE Guides of the Australian Commission on Safety and Quality of Healthcare [4]. Over the last decade, the role of teamwork and coordination at the healthcare level has gained prominence as it has been shown to bring a variety of perspectives, experiences, and competencies, which strengthen the continuity of patient care [5].

Communication is an essential mechanism in collaboration and a key element in the success of organizations [6] as it is present in practically all the processes carried out in a healthcare center and is an increasingly valued competence [7]. However, despite its relevance, communication continues to be presented as a problem and a challenge at the care level and is still included as one of the objectives in the “National Patient Safety Goals” (NPSGs) [8] and “International Patient Safety Goals” (IPSGs) [9].

According to the WHO, a large proportion of adverse events occurring in healthcare services worldwide are due to communication failures [10]. In fact, the Joint Commission highlights that lack of communication between professionals is a factor in 80% of all adverse events [11]. In Spain, the “Patient Safety Strategy” highlights the transfer of patients from one professional to another (“handoff” or “handover”) as one of the most critical moments in the communication process [12], which is an essential and frequent part of nursing work due to continuous shift changes and constant contact with other professional groups [13]. Carrying out the handoff process appropriately and effectively is a challenge [2]. For example, a qualitative study highlighted four main elements that impact the quality of interprofessional communication: the environment, interpersonal relationships, personal factors, and lack of training [14]. Among these, the hierarchical nature of medicine is a very relevant factor [5,15], as when there are no established protocols, guidelines, or tools for communication, decision-making or conflict resolution is more likely to be determined by factors such as hierarchy or years of experience [16]. In addition, differences in communication style between nursing and medical staff have been cited as one of the elements that hinder interprofessional communication in the healthcare setting [2,17], as the holistic approach of nursing clashes with the detail-oriented approach of medicine [18]. Communication should be enhanced not only at the oral level, but also at the level of written messages. A study in an internal medicine unit analyzed text messages sent urgently between nurses and doctors in deteriorating patients requiring ICU admission. In this study, only 3% of the messages included all components of the SBAR, pointing to an acute need for improved communication between professionals [19]. In this sense, nurses found the SBAR to be a very useful tool that improved patient safety and teamwork [20,21]. In addition, another study pointed out how SBAR is acceptable to emergency medicine residents as it facilitates communication in the clinical setting between physicians and residency training programs [22]. Consequently, clarity in communication processes is necessary, which can be achieved through SBAR; this has been shown to improve hospital survival [19].

Improving communication between professionals is crucial and could prevent millions of adverse events [10]. To this end, it is necessary to ensure that the information shared is accurate and appropriate, a goal that can be achieved by standardizing communication processes, especially during the transition of care, as this minimizes message variability and increases the effectiveness of communication [12].

### The Impact of Communication on Professional Wellbeing

Stress and fatigue are elements frequently present in the healthcare environment, especially associated with nursing [23], and are highly related to burnout, which decreases productivity, efficiency, and quality of care [24]. Although poor communication in an organization and among its employees is one of the factors that most affects staff dissatisfaction [6], there are few studies that have analyzed the impact of interprofessional communication on the well-being of healthcare professionals [25]. The present research aims to precisely relate these concepts from the positivist psychology model in the field of occupational health. Therefore, the well-being of workers is assessed through indicators of capabilities and strengths related to emotional intelligence, such as job satisfaction, engagement, and resilience.

Job satisfaction can be defined as the degree to which a person is satisfied with the intrinsic and extrinsic characteristics of the job [26]. Engagement is defined as “a positive and enriching work-related state of mind characterized by vigor, dedication, and absorption” [27]. In the workplace, engagement has gained popularity for its key role in combating burnout in healthcare professionals, which links it closely to job satisfaction [27,28]. In fact, there is evidence that engagement is the strongest and most significant predictor of job satisfaction [29]. In this sense, the implementation of the SBAR technique in a neonatal obstetrics unit has increased workers’ job satisfaction. It has also improved communication, teamwork, and safety between doctors and nurses [20]. However, to date, no studies have been found relating SBAR to staff satisfaction in internal medicine units. This is of interest, because a study conducted on internal medicine residents reported a reduced level of well-being associated with low job satisfaction due to major medical errors, suicidal ideation, and a desire to leave clinical practice [30]. Furthermore, it has also been observed that job satisfaction among IM nurses can be improved by a favorable working environment; this is because satisfaction with colleagues and with their immediate bosses is one of the most highly rated aspects [31].

Another concept closely linked to the previous ones is resilience. According to the American Psychological Association (APA), it is the process of adapting well to adversity, trauma, tragedy, threat, or significant sources of stress [32]. It is of particular relevance in healthcare organizations, as developing resilience is considered one of the effective methods to reduce stress and burnout [33] and to improve professional qualities and job satisfaction, thus reducing cases of depression and job burnout [34]. To develop resilience, it is essential that risk factors exist alongside protective factors that reduce or eliminate their negative effects. One such factor is communication skills, which is a key tool for increasing levels of resilience [34], and therefore, engagement and job satisfaction. In fact, the American Medical Association (AMA) has recommended improving communication and teamwork as strategies to reduce burnout among healthcare professionals [35].

The SBAR method has been widely used in neonatal [36], pediatric [7], and obstetric units [20]. Its advantages include improved communication, trust, quality of care for the patient, and safety [36]. The SBAR method has been used by nurses when changing shifts in coronary units with positive results in increasing the quality of care received by the patient with improved management of the patient’s condition, including anxiety, anger, and loneliness [37]. Additionally, SBAR is associated with a reduction in unexpected deaths and an increase in unplanned ICU admissions [38]. In this regard, lack of communication is common in healthcare settings and is especially important in-patient transfers and in areas where fast and efficient management is needed [2]. Despite the above, no studies have been found implementing the SBAR methodology within internal medicine units to improve the well-being of workers. Thus, the aim of this research is to evaluate the impact of the use of the SBAR method for improving communication on the degree of job satisfaction, engagement, and resilience of healthcare staff in the internal medicine unit of a university hospital in Castilla y Leon (Spain).

## 2. Materials and Methods

### 2.1. Type of Study

This quasi-experimental study was an observational, descriptive, longitudinal study with pre- and post-implementation analysis of the SBAR method. We chose to approach the work as a single case study because, as Sandelowski [39] indicated, the structure of a case study in research represents the holistic nature of nursing.

### 2.2. Study Unit

This study was carried out in the Internal Medicine Unit of a regional hospital in Castilla y León (Spain), in control A (one of the two sub-units of the ward), made up of a team of auxiliary nursing care technicians, nurses, and doctors.

The pre-intervention was carried out among the unit’s staff, with a total of 41 workers, of whom 10 were auxiliary nurses, 16 were nurses, and 15 were physicians; 36 participated in the study. Due to staff changes in the unit because of relocations or sick leave, it was sometimes necessary to have substitute staff present with the same characteristics in terms of shifts and timetables, to whom the intervention was explained once again. Thus, the post-intervention sample consisted of a total of 40 workers, of whom 11 were auxiliary nurses, 15 were nurses, and 14 were physicians, of whom 35 subjects participated in the study. No differences were found between groups for the demographics of gender, professional category, or length of service with respect to pre- and post-intervention.

### 2.3. Study Variables

The socio-demographic variables used to describe the sample were:

- Sex: male and female.
- Professional category: auxiliary nurse, nurse, and doctor.
- Seniority of service: less than 10 years and equal to or greater than 10 years.

### 2.4. Instruments

The Overall Job Satisfaction Scale, developed by Warr, Cook, and Wall [26], or the General Job Satisfaction Scale NTP 394 in its Spanish version adapted by Pérez Bilbao and Fidalgo [40], was used to measure job satisfaction. It is made up of 15 items (the items of this questionnaire can be seen in Section 3) and consists of two subscales, one of extrinsic factors (8 items, with Cronbach alpha ranged from 0.74 to 0.78) and the other of intrinsic factors (7 items with Cronbach alpha ranged from 0.79 to 0.85). The response format was a 7-point Likert-type scale, where 1 is very dissatisfied and 7 is very satisfied with α ranging from 0.85 to 0.88. The total score was obtained by adding the values of all the items, which ranges from 15 to 105; the higher the score, the higher the overall satisfaction.

The Utrecht Work Engagement Scale (UWES 17) was used [27], which is a 17-item questionnaire that explores the three dimensions of engagement through three subscales: vigor (6 items), dedication (5 items), and absorption (6 items) (the items of this questionnaire can be seen in Figure 2). Each item was answered on a scale from 0 (never) to 6 (always). The total score was obtained by adding the score and then dividing by the number of items, so the range was between 0 and 6, with five levels of total score: very low (<1.93), low (1.94–3.06), medium (3.07–4.66), high (4.67–5.53), and very high (>5.54). Cronbach’s alpha values for the 17-item scale typically range between 0.91 and 0.96 [27].

Resilience was measured with the Connor Davidson Resilience Scale (CD-RISC). The original scale consists of 25 items, and the reduced 10-item scale [41] validated in Spanish in 2011 by Notario Pacheco et al. [42] was used for this work (the items of this questionnaire can be seen in Figure 3). Respondents give each item a value from 0 (never) to 5 (always). The score ranges from 0 to 40 points, so that the higher the score, the greater the resilience. Cronbach’s alpha in the Spanish population was 0.85 [42].

### 2.5. Procedure

The questionnaires were delivered to the unit staff in March 2016, obtaining data on the variables prior to the implementation of SBAR in the plant. From November 2016 to January 2017, the intervention was carried out, with follow-up visits to ensure the implementation of the SBAR method by the professionals. The application of the SBAR method consists in the improvement of communication by means of organizing information according to acronyms [43]: (a) situation: description of the problem; (b) background: background information related to the current problem; (c) assessment: evaluation and opinion of the cause of the problem; (d) recommendations: recommendations or suggestions for the patient’s plan. Finally, the questionnaires were distributed again and collected in January 2017 to assess the variables after the intervention.

### 2.6. Data Analysis

Statistical analysis was carried out with the IBM^®^ SPSS^®^ v.26 program. First, descriptive statistics were obtained to define the sample (mean, standard deviation, percentages, and maximum and minimum). Secondly, an independent group *t*-test and an ANOVA test were performed to assess the differences between the interventions and the scales with the different variables. In addition, Pearson’s correlation was performed to analyze the relationship between the different scales, all after having checked the normal distribution of the sample with the Kolmogorov-Smirnov test, obtaining a significance level > 0.05 in all the variables studied.

### 2.7. Ethical Aspects

This study was approved by the manager of the hospital by means of an ethical protocol, which guarantees compliance with all ethical and legal aspects. The treatment of the information and the development of the work was done respecting confidentiality at all times in accordance with the Organic Law 3/2018 of 5 December on Personal Data Protection and guarantee of digital rights.

## 3. Results

### Description of the Sample and Socio-Demographic Data

The questionnaire was answered by 36 individuals in the pre-intervention (with a participation rate of 87.8%) and by 35 individuals in the post-intervention (with a participation rate of 87.5%).

The results obtained for each of the socio-demographic variables can be seen in Table 1.

**Figure 1:**
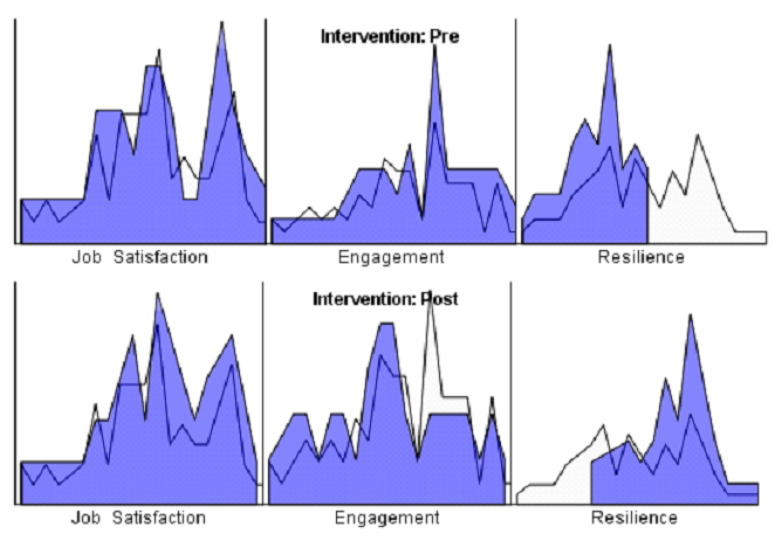
Results obtained before and after application of the SBAR method.

As shown in Table 2, a student’s *t*-test was performed to compare the means of the pre- and post-intervention scores, and descriptive results are included for each of the study variables (job satisfaction, engagement, and resilience). Figure 1 shows the different results obtained for both pre- and post-intervention.

As a result of the Student’s *t*-test for the differences in means between the pre- and post-intervention, resilience values were significantly higher in the post-intervention (*X* = 38.46 ± 4.62) than in the pre-intervention (*X* = 28.03 ± 3.96), (*p* = 0.000).

A Pearson correlation was performed between the three scales used to assess the association between them, finding in both interventions a statistically significant moderate direct association between job satisfaction and engagement (*p* = 0.006, r = 0.450 in the pre-intervention, and *p* = 0.000, r = 0.063 in the post-intervention).

After performing Student’s *t*-test for equality of means and an ANOVA between job satisfaction and the different variables in the sample, a significant difference (*p* = 0.035) was found in the professional category variable between medical and nursing staff, with doctors obtaining the best scores, with an average of 74.29 ± 11.95, and the group of nurses the worst, with an average of 60.00 ± 15.26.

In short, the professional category with the lowest job satisfaction scores on each of the scales in both the pre- and post-intervention was that of nurses, and the only significant differences found were between the group of doctors and nurses in the pre-intervention.

**Figure 2:**
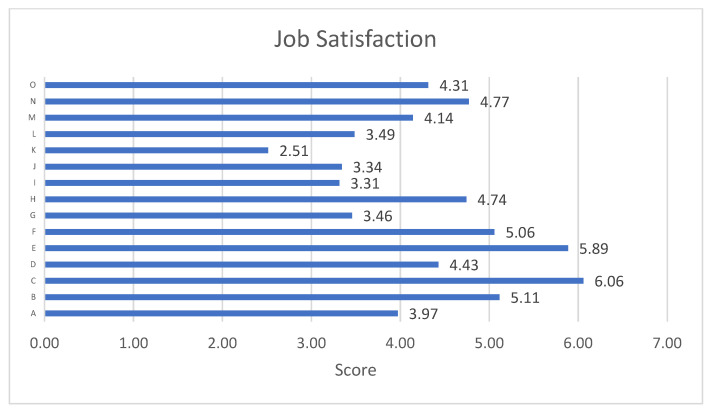
Scores on the dimensions of the overall job satisfaction scale at post-intervention. Note: A: Physical working conditions. B: Freedom to choose your own working method. C: Colleagues at work. D: Recognition for work well done. E: Immediate superior. F: Assigned responsibility. G: Salary. H: The possibility to use your capabilities. I: Management-worker relations. J: Chances of promotion. K: The way in which the company is run. L: Attention to suggestions. M: Working schedule. N: The variety of tasks performed. O: Job stability.

The post-intervention data obtained in each item of the general job satisfaction scale are shown in Figure 2. The item providing the highest job satisfaction was C: your co-workers, followed by E: your immediate superior, while the items with the worst satisfaction averages were K: the way your company is managed, and I: management-worker relations in your company.

Significant differences were obtained in terms of the engagement values and the sex of the participants both in the pre- (*p* = 0.014) and post-intervention (*p* = 0.004), with males showing a higher level of engagement in both cases (*X*pre = 5.01 ± 0.54 and *X*post = 5.03 ± 0.71) than females (*X*pre = 4.06 ± 0.92 and *X*post = 3.72 ± 0.88). No other significant relationships were found between the other variables and the level of professional engagement.

**Figure 3:**
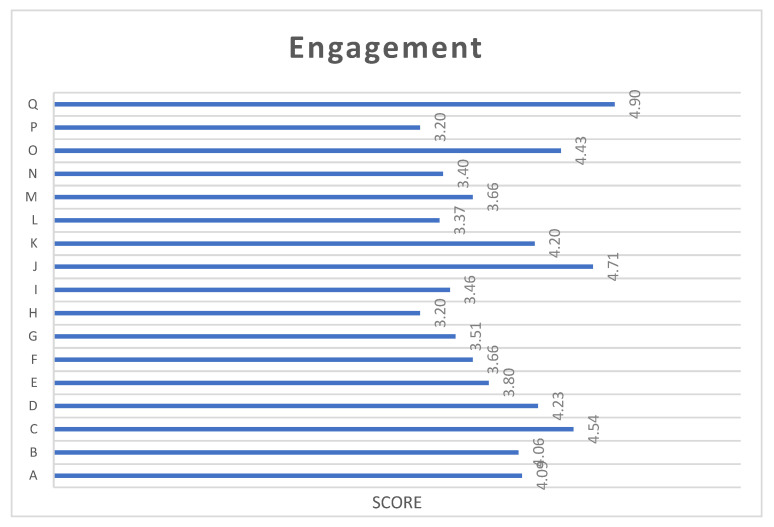
Scores on the dimensions of the UWES-17. (Note: A: In my work I feel full of energy, B: my work is full of meaning and purpose, C: time flies when I am working, D: I am strong and vigorous in my work, E: I am enthusiastic about my work, F: when I am working I forget everything that is going on around me, G: my work inspires me, H: when I get up in the morning I feel like going to work, I: I am happy when I am absorbed in my work, J: I am proud of the work I do, K: I am immersed in my work, L: I can continue to work for long periods of time, M: My work is challenging, N: I get carried away by my work, O: I am very persistent in my work, P: I find it difficult to disconnect from my work, Q: when things go well, I continue working).

The results obtained in the post-intervention for each of the dimensions of the UWES-17 scale can be seen in Figure 3, in which it can be observed that the item with the highest score was Q: when things go well, I continue working, and the lowest score was H: when I get up in the morning, I feel like going to work.

Regarding the resilience values, it should be noted that the nursing staff obtained the highest resilience values (*X*pre = 29.42 ± 3.98 and *X*post = 41.23 ± 3.89) of all the professional groups, while the auxiliary nurse group obtained the lowest values (*X*pre = 26.30 ± 3.74 and *X*post = 35.82 ± 3.71), leaving the physician group in an intermediate position (*X*pre = 28.07 ± 3.89 and *X*post = 37.82 ± 4.99).

The only significant differences (*p* = 0.008) were those obtained in the post-intervention between the nursing staff (*X* = 41.23 ± 3.89) and the auxiliary nursing care technicians (*X* = 35.82 ± 3.37).

**Figure 4:**
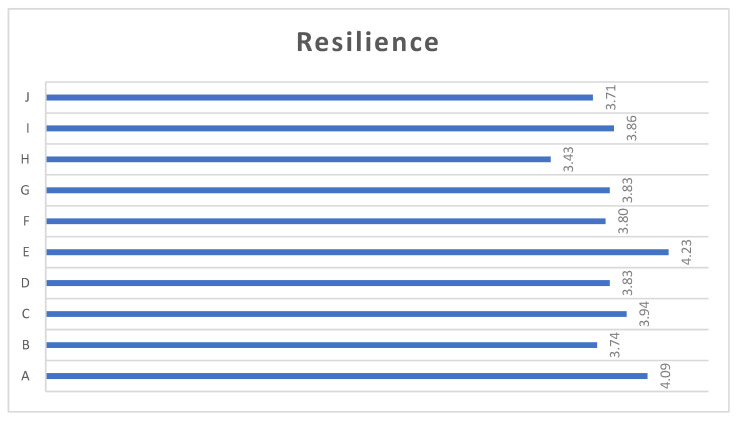
Scores on the dimensions of the CD-RISC 10 scale. (Note: A: I can adapt to change, B: I can handle any situation, C: I see the positive side of things, D: I can handle myself well in spite of pressure or stress, E: After a serious setback I usually “bounce back”, F: I manage to achieve my goals in spite of difficulties, G: I can keep my concentration under pressure, H: I hardly get discouraged by failures, I: I define myself as a strong person, J: I can handle unpleasant feelings.).

The results of each item of the CD-RISC scale for post-intervention are shown in Figure 4, which shows that there are no major differences between the scores given to each of the items.

## 4. Discussion

The aim of this study was to evaluate the impact of implementation of the SBAR method to improve communication on the degree of job satisfaction, engagement, and resilience of healthcare staff in the internal medicine unit of a university hospital in the province of Leon (Spain).

In relation to job satisfaction, the results obtained both pre- and post-intervention are at an average, which can be considered a positive result if we take into consideration that internal medicine services, being characterized by a greater workload and highly complex acute patients, have higher indicators of burnout and emotional exhaustion and a lower degree of job satisfaction among their workers when compared with other services [44].

Our results suggest that the SBAR intervention does not seem to have a significant impact on job satisfaction values. However, a study conducted in an emergency department in the USA found an increase in job satisfaction among nurses after the implementation of SBAR together with other tools to improve teamwork, such as joint patient assessment and information sharing meetings [45]. Similarly, another study in Taiwan found an improvement in teamwork and job satisfaction among nurses after starting to use the SBAR method in a maternity ward [20]. Likewise, quasi-experimental research study carried out in a hospital in Indonesia suggested that one of the essential factors for improving job satisfaction is effective communication; a comparison of the job satisfaction of nurses before and after the implementation of SBAR showed a significant increase after the intervention, thus concluding that SBAR is a useful tool to achieve an improvement in job satisfaction among nurses [46]. SBAR has increased nurses’ job satisfaction in intensive care units [47]. However, no studies have been found where all team members were included. Thus, improving communication between professionals could avoid adverse events [10]. It has been shown that the use of communication enhancement tools such as SBAR and the optimization of text messaging capabilities within clinical messaging systems could improve rapid response activation and the quality of communication between nurses and physicians [19].

However, communication is one of the factors influencing job satisfaction, others being: autonomy level, working conditions, respect and recognition for the work performed, adequate and sufficient staff, good relations between team members, salary, commitment to the organization, and professional involvement, with many aspects being related to the hospital’s management style and model [48]. Given these considerations, it can be assumed that the impact of SBAR may have been limited by other aspects of ward work, preventing it from being reflected in job satisfaction levels.

Regarding the relationship of job satisfaction with socio-demographic variables, in our analysis, job satisfaction was affected only by professional category, with the group of doctors scoring significantly higher than nurses or auxiliary nursing care technicians in the pre-intervention. These inequalities are reduced in the post-intervention, as no significant difference is obtained. Previous studies have found that the relationships between job satisfaction and professional category are contradictory. In a study carried out in primary healthcare centers in area V of Asturias [48], the nursing category had a higher percentage of satisfaction than the medical group. Furthermore, the literature points to significant differences in terms of gender and professional category: male physicians show higher job satisfaction scores than the nursing group, while female physicians have the worst job satisfaction scores of all professional groups [25]. In contrast, in an Iraqi study, women scored higher than men on Warr Cook and Wall’s overall job satisfaction scale [49]. Moreover, in a study on the implementation of SBAR in India, 79% of nurses expressed that they found SBAR to be a very useful tool as it allowed them to organize all important information in a simple way, thus improving patient safety [21]. This reinforces the idea that job satisfaction depends on different factors, as there is considerable variability in the results depending on the sample

Regarding the items of the job satisfaction scale, in the post-intervention of our study, co-workers and the immediate superior stand out as the elements best valued by the professionals, which is consistent with what was obtained in another study [48], where the dimensions of greatest satisfaction were the “interpersonal relationship with co-workers” and the “interpersonal relationship with bosses”. In addition, the lowest rated items were “the way your company is managed” and “management-worker relations in your company”, both of which are related to management. This could be key to understanding the results obtained, as many of the factors that influence job satisfaction are related to the organizational environment and it is precisely these items that are the lowest rated in the questionnaire.

Turning our attention to engagement, this construct is considered to be the strongest and most significant predictor of job satisfaction [29]. The values obtained in our sample correspond to the medium range of the manual [27], positive results similar to those of a study that assesses the levels of engagement and resilience among nurses responsible for patients with COVID-19 in China, in which, with these results, they conclude that their engagement is high [50]. As for the relationship with the sociodemographic variables, there is a clear effect of gender on the levels of engagement throughout the research, with men’s engagement being higher (*p* = 0.014 in the pre-intervention and *p* = 0.004 in the post-intervention). This relationship is already reported in the UWES scale manual, in which higher scores are obtained for men than for women [27]. In a study of a sample of hemodialysis nurses, men also have higher levels of engagement than women, and nurses with more experience and years of work have higher scores [51].

Finally, the resilience values obtained in the pre-intervention of the present study coincide with those of a Spanish study [52] that analyses the levels of resilience using the 10-item CD-RISC scale in a sample of doctors, nurses, and auxiliary nursing care technicians in an ICU in Madrid. However, after the implementation of the SBAR, the level of resilience of our sample increased notably, moving away from the results of the Madrid study. This increase is in line with an Australian study [53], which developed a communication-based intervention to improve resilience in a sample of nurses and midwives. It concludes that, following the intervention, there was an increase in all resilience-related parameters and a change in communication style to a more professional and assertive one, improving the work environment. Gerhart et al. [28] also obtained significant results in improving staff resilience and engagement in a palliative care facility, where, in addition, symptoms and indicators of depression, depersonalization, and post-traumatic stress among staff decreased.

In our study, it stands out that nursing staff was the professional group with the highest resilience values in the sample, which coincides with Lyu et al. [50] who obtained very high levels of resilience among frontline nurses in the fight against COVID-19, with an average of three points above the normative scores of the scale used.

This is particularly relevant given the key role resilience plays in the occupational well-being of professionals. As research on hemodialysis nurses concluded [51], resilience is the most powerful tool for improving levels of engagement, which is in agreement with another study that shows a significant and positive relationship between resilience and engagement, dedication, concentration, and energy at work [50].

Despite the above, the present study has some limitations that need to be pointed out. Firstly, the sample is considerably small and was selected for convenience, which makes it difficult to generalize and extrapolate the results. Moreover, although this is common in the healthcare field, especially in nursing, the fact that most of the sample was female is a factor to be considered. In addition, it should be noted that the intervention took place between 2016 and 2017, which was when the communication protocol was established in the unit. Longitudinal studies are needed, which also analyze the consequences or effects of improved communication at the user level, with measurements of mortality rates, length of admission, and other variables, plus the subjective wellbeing of workers.

## 5. Conclusions

The levels of all variables studied were in average or even high ranges compared to normative scores and other studies. After the implementation of the SBAR method, the levels of resilience increased considerably among the staff. However, a slight decrease in levels of job satisfaction and engagement was also observed among professionals. The nursing professional group stands out as the one with the lowest job satisfaction of all, although the gap with the other professional groups decreases after the intervention. In contrast, nurses have the highest levels of resilience of all professional groups. This may be due to staff changes in the unit and the integration of rotating staff between various units.

After obtaining these results, it should be a priority to ensure the correct implementation of the SBAR method, assessing whether its use by workers is adequate and considering that SBAR is frequently studied in conjunction with strategies to facilitate its implementation, elements that could be improved.

Another priority task would be to assess whether there were any major stressors during the intervention year that may interfere with the introduction of SBAR. In this respect, it is worth noting that the lowest rated items of the post-intervention job satisfaction scale were related to the organizational environment of the hospital. In addition, the demographic analysis of the sample showed a very significant decrease in the percentage of professionals with a permanent employment contract in the post-intervention period compared to the pre-intervention period, an issue that coincides with the precariousness of the sector that has taken place in recent years, with the consequences that this entails for the health and well-being of the workers.

## Figures and Tables

**Table 1:** Sociodemographic descriptions of the sample.

			Pre-Intervention (n = 36)	Post-Intervention (n = 35)
Male	Physician	<10 years	4 (57.1)	2 (40.0)
		>10 years	3 (42.9)	3 (50.0)
Female	Physician	<10 years	5 (71.4)	4 (66.7)
		>10 years	2 (28.6)	2 (33.3)
	Nurse	<10 years	7 (58.3)	7 (53.8)
		>10 years	5 (41.7)	6 (46.2)
	Auxiliary nursing care technicians	<10 years	8 (80)	9 (81.8)
		>10 years	2 (20)	2 (18.2)

**Table 2:** Variations of the variables between the two interventions by the Student’s *t*-test.

	Pre-Intervention		Post-Intervention		Range	*p*
	Mean ± SD	Max/Min	Mean ± SD	Max/Min		
Job satisfaction	66.39 ± 15.00	92/31	64.60 ± 13.97	91/33	15–105	0.143
Engagement	4.24 ± 0.93	5.88/1.94	3.90 ± 0.97	5.71/2.00	0–6	0.605
Resilience	28.03 ± 3.96	35/18	38.46 ± 4.62	49/28	0–40	0.000

## Data Availability

Data from this study will be made available upon reasoned request to the corresponding author.
